# Associating rare genetic variants with human diseases

**DOI:** 10.3389/fgene.2015.00133

**Published:** 2015-04-08

**Authors:** Qunyuan Zhang

**Affiliations:** Division of Statistical Genomics, Washington University School of MedicineSt. Louis, MO, USA

**Keywords:** rare genetic variants, human diseases, association, statistical analysis methods, burden tests, non-burden tests

Genome researches have revealed that a large portion (over 50%) of genetic variants on human chromosomes are rare variants (RVs) with extremely low allele frequency (usually defined as less than 1%) in populations. In recent years, advances of DNA genotyping and sequencing technologies have been facilitating the discovery of RVs, and the association between RVs and human diseases is of rapidly growing interest in understanding genetic and molecular mechanism of both common and rare diseases (Cirulli and Goldstein, 2010; Gibson, 2012).

As a potential source of contribution to the “missing heritability” that cannot be explained by common variants in most SNP-array based genome wide association studies (GWAS), RVs have been identified to be associated with some human diseases or disease-related complex traits, such as cholesterol level (Cohen et al., 2004), hypertriglyceridemia (Johansen et al., 2010), autoimmune disease (Hunt et al., 2012), and Alzheimer's disease (Lord et al., 2014). In spite of these findings, it is still quite challenging to identify the RV association for many diseases and traits, due to the rarity of RVs in populations.

When allele frequencies become very low, the odds of observing RVs in study samples will be small. As a result, the lack of variation in the observed data usually makes the statistical test of association significantly underpowered, which has been widely recognized as the major issue in most RV association studies. To improve the power, numerous statistical methods have been developed, investigated, and used in recent years (Bansal et al., 2010; Basu and Pan, 2011; Ladouceur et al., 2012; Dering et al., 2014; Lee et al., 2014), mostly based on a hypothesis that multiple RVs within a genetic unit (usually a gene) may function in a similar way and collectively contribute to a disease (such phenomena have been observed in many diseases). These methods can be categorized into burden test, non-burden test, and unified test. When burden tests, e.g., CMC (Li and Leal, 2008) and WSS (Madsen and Browning, 2009), are designed for detecting the association of a genotypic “burden” score summarized from a set of RVs, non-burden tests keep individual RVs as individual variables and evaluate whether at least one of multiple RVs is associated with the trait (Wu et al., 2011; Kim et al., 2014; Wang, 2014), and unified tests provide a hybrid analysis combining both burden and non-burden tests (Lee et al., 2012; Sun et al., 2013). In general, burden tests are more powerful when the portion of causal variants increases, non-burden tests may outperform when only a small portion of variants are causal, and unified tests provide an optimized balance between burden and non-burden methods (Lee et al., 2012). One key feature of these methods is that they test the collective effect (not individual effects) of multiple RVs as an entire group, therefore, once the association of a group of RVs is identified, further analyses are still required to determine which one or ones in the group cause the association. Another limitation is that although these methods provide useful tools for association analysis, they usually lack the estimation of heritability of RVs; further analyses of heritability using appropriate methods, e.g., bootstrap-sample-split algorithms (Liu and Leal, 2012), are still warranted.

The main benefit from collective tests is that statistical power can be substantially increased if the majority (or a significant portion) of a set of RVs have effects on a trait. However, the power can be compromised by neutral or “noise” variants (i.e., the variants with no effects) mingled in analysis. To deal with this issue, many bioinformatics databases and tools (Adzhubei et al., 2010; Wang et al., 2010; Preeprem and Gibson, 2014) have been used to annotate or predict the functions of RVs, and the variants that are unlikely to have functions (e.g., synonymous variants) are usually excluded. For those RVs kept in an analysis after the exclusion based on bioinformatics prediction, since their contributions to a trait could be very different, many methods have been proposed to assign different weights to individual RVs when building a collective test. Weights for individual RVs can be determined by, for example, allele frequency (Madsen and Browning, 2009), genotyping score, annotation score, and many statistics or probabilities estimated from the experimental and/or public data. Particularly, adaptive weighting methods (Lin and Tang, 2011; Pan and Shen, 2011; Zhang et al., 2011) even allow weights to be learned from an initial association analysis using the observed genotype and phenotype data; usually, these methods can improve power at a cost of computational burden, especially when sample sizes are large.

In a collective test, because RVs are tested set by set (not variant by variant), how to select a RV set is critical and will significantly affect the power. Although typically RVs are grouped and selected by gene, there are many other ways to determine the RV sets, for example, by chromosomal region, by exon, by functional pathway, etc. The allele frequency cutoff can be fixed (e.g., <0.01) or a variable threshold method (Price et al., 2010) can be adopted. Since there is no a gold standard for RV grouping and selection, in practice it may need to try multiple ways based on different hypotheses to optimize an analysis.

One of the most popular experiment designs for the RV association analysis is to compare two groups of subjects, such as case and control groups, or two groups differentiating in a quantitatively measurable trait (e.g., low vs. high blood pressure). Analysis of data from such designs can be performed in either a simple way that tests the enrichment of RVs in one group (vs. another group), or a more sophisticate way that models the group assignment as a binary outcome variable dependent on individual and/or summarized RV variables as well as other confounding covariates (e.g., age and gender) if necessary. Although most designs and statistical methods were originally introduced for binary outcomes from unrelated subjects, many of them have been extended to quantitative traits and family data (Fang et al., 2012; Chen et al., 2013; De et al., 2013; Ionita-Laza et al., 2013; Zhang et al., 2014).

Since the potential of power improvement via statistical methods is limited, the ultimate and most effective way of increasing power yet relies on larger sample sizes, which can be realized by either increasing the sample size in a single experiment, or combining the results from multiple experiments into a meta-analysis using recently developed methods (Hu et al., 2013; Lee et al., 2013; Feng et al., 2014; Liu et al., 2014). Once an adequate power is achieved and a significant, solid statistical association between RVs and a disease has been identified, the associated RVs can be very useful in many ways, such as biomarker design, biological function validation, molecular mechanism discovery, drug development, etc.

## Conflict of interest statement

The authors declare that the research was conducted in the absence of any commercial or financial relationships that could be construed as a potential conflict of interest.
